# PRIMA-1 and PRIMA-1^Met^ (APR-246): From Mutant/Wild Type p53 Reactivation to Unexpected Mechanisms Underlying Their Potent Anti-Tumor Effect in Combinatorial Therapies

**DOI:** 10.3390/cancers9120172

**Published:** 2017-12-16

**Authors:** Anne Perdrix, Ahmad Najem, Sven Saussez, Ahmad Awada, Fabrice Journe, Ghanem Ghanem, Mohammad Krayem

**Affiliations:** 1 Laboratory of Oncology and Experimental Surgery, Institut Jules Bordet, Université Libre de Bruxelles, 1 rue Heger-Bordet, 1000 Brussels, Belgium; anne.perdrix@chb.unicancer.fr (A.P.); anajem@ulb.ac.be (A.N.); ahmad.awada@bordet.be (A.A.); fabrice.journe@bordet.be (F.J.); gghanem@ulb.ac.be (G.G.); 2 Clinical Laboratory, Department of Biopathology, Henri Becquerel Centre, 76038 Rouen, France; 3 Equipe de Recherche en Oncologie (IRON), Inserm U1245, Rouen University Hospital, 76000 Rouen, France; 4 Laboratory of Human Anatomy and Experimental Oncology, Research Institute for Health Sciences and Technology, University of Mons (UMONS), 7000 Mons, Belgium; sven.saussez@umons.ac.be; 5 Department of Medicine, Institut Jules Bordet, Université Libre de Bruxelles, 1000 Brussels, Belgium

**Keywords:** p53, p53 reactivation, PRIMA-1, PRIMA-1^Met^, APR-246, drug combination, cancer

## Abstract

p53 protects cells from genetic assaults by triggering cell-cycle arrest and apoptosis. Inactivation of p53 pathway is found in the vast majority of human cancers often due to somatic missense mutations in TP53 or to an excessive degradation of the protein. Accordingly, reactivation of p53 appears as a quite promising pharmacological approach and, effectively, several attempts have been made in that sense. The most widely investigated compounds for this purpose are PRIMA-1 (p53 reactivation and induction of massive apoptosis )and PRIMA-1^Met^ (APR-246), that are at an advanced stage of development, with several clinical trials in progress. Based on publications referenced in PubMed since 2002, here we review the reported effects of these compounds on cancer cells, with a specific focus on their ability of p53 reactivation, an overview of their unexpected anti-cancer effects, and a presentation of the investigated drug combinations.

## 1. Introduction

p53, so called “the guardian of the genome“, appears as a key factor in the carcinogenesis. Indeed, somatically, TP53 is the most frequently mutated gene in human cancer overall [1]. The outcome of mutations in the tumor suppressor gene p53 results in the loss of the wild-type p53 (wt-p53) activity and the gain of oncogenic functions such as resistance to apoptosis and escalation in genome instability. Indeed, cell cycle control, senescence, apoptosis, and DNA repair are deregulated due to mutations in p53. In addition, such mutations push cancer cells to acquire new properties, promoting invasion, migration, angiogenesis, proliferation, genomic instability, or drug resistance [2]. Mutant-p53 are, consequently, associated with aggressive tumor phenotypes and poor patient survival.

For the two last decades, therapeutic strategies to enhance or to restore p53 activity appeared as a major domain of the anticancer research. Two strategies have been used. The first one involves pharmacological restoration of wild type p53 by prevention of MDM2/4(Mouse double minute 2) homolog mediated proteasomal degradation. Several classes of molecules that activate wild p53 by targeting MDM2/4 or other upstream regulators of p53 have been reported. These molecules and their different mechanism of action were discussed by Selivanova [3,4]. The nutlins were the first discovered molecules of this class. Cell-based screens to find further inhibitors of the p53-MDM2 interface lead to the identification of RITA (reactivation of p53 and induction of tumor cell apoptosis) [3]. RITA activity directly correlates with the ability to induce DNA damage. RITA is not dependent on MDM2 but is a genotoxic compound (previously known as NSC652287) and according to p53 status, it induces a p53-dependent or independent cell death [5].

The second strategy to reactivate p53 consists of restoring its active conformation. Over the last decade, several attempts have been made to identify compounds that are able to reverse the oncogenic properties of mutant p53. Boecker et al., 2008 [6] focused their studies on a specific hot mutation in p53 (Y220C) and they designed Y220C-targeting compounds based on in silico analysis of the crystal structure of the p53 core domain including PK083 and PK7088. These molecules have shown to induce Y220C-dependent apoptosis in tumor cells. Bykov et al. [7] screened a library of 2000 low-molecular-weight compounds from the National Cancer Institute using a cell-based assay in order to identify compounds that can restore wild-type function to mutant p53. This screening has led to the identification of the molecule 2,2-*bis*(hydroxymethyl)-1-azabicyclo[2,2,2]octan-3-one, named PRIMA-1 for “p53 reactivation and induction of massive apoptosis”. Since 2002, PRIMA-1 and its structural analog PRIMA-1^Met^, named ‘APR-246’, have been studied in several models, alone or associated with other cancer treatments.

The aim of this review, based on the publications referenced in PubMed since 2002 and using the key words PRIMA-1 or APR-246, is (1) to question, 15 years after their discovery, the initial published properties of PRIMA-1 and APR-246 (cancer growth inhibition and mutant-p53 reactivation), (2) to present the new hypothetical mechanisms of action of these molecules, and (3) to list and comment the therapeutic associations already tested and ongoing, in order to prepare the future clinical combination of p53 restoration and targeted therapies.

## 2. Effects of PRIMA-1 and APR-246 on Cancer Growth Inhibition and Mutant-p53 Reactivation

At the beginning, Bykov et al. (2002) [7] screened compounds that could suppress the proliferation of human tumor cells harboring mutation in p53. Since this first publication, many studies have reported the tumor suppressor effects of PRIMA-1, then APR-246, in various cancers. Indeed, APR-246, the structural analog PRIMA-1^Met^, has been presented since 2005 as more active than PRIMA-1 [8] with superior permeability properties [9]. This molecule has gradually become as studied as PRIMA-1. In this review, we reported the results published using the two compounds. We presented the conclusions established with either PRIMA-1 or APR-246 using the name PRIMA-1/APR-246. For specific antitumor effect of one compound, we specified the tested molecule and presented the specific studies in Table 1.

The evaluated tumor types were: lung cancer [8,10,17,20,21,22,24,25,27,34,46,59,62,65], ovarian [35,40,51,52,65] and colorectal cancer [20,21,22,24,42,56,65], leukemia [11,13,30,53,63], breast cancer [12,14,15,21,23,28,33,45,61,66], sarcoma [8,20,22,24,34,41,57,65], prostate cancer [18,49], liver cancer [19,67], head and neck cancer [26], malignant melanoma [29,58,59,64], bladder cancer [44,68], thyroid cancer [31,38,39,47], myeloma [36,37,54,55], pancreatic cancer [32,67], Waldenström macroglobulinemia [43], glioblastoma [60] and oesophageal cancer [48,62]. Most of these studies used established cell lines to evaluate the PRIMA-1/APR-246 antitumor capacities, but primary cultures [4,11,13,30,36,37,40,43,51,53,54,57], especially for hematological malignancies [11,13,30,37,43,53,54], and xenografts [8,19,27,28,29,36,37,38,40,42,48,49,55,56,58,62,65] have also been assessed to validate the in vitro data and to evaluate toxicity.

The effect of PRIMA-1/APR-246 on the inhibition of cell proliferation and vitality, have been documented in vitro using tetrazolium salts based tests [8,17,24,25,27,31,32,38,39,41,43,44,54,55,56,60,63,65], sulforhodamine assay [50], sulforhodamine assay [62], vital stainings [12,19,26,47,48,58,60,66], fluorometric microculture cytotoxicity assay [51], or the measurement of intracellular ATP content [11,13,42,52,53]. The inhibition of colony formation [8,18,26,32,36,42,43,48,56,60,61] and cancer cell migration [36,42,43] also highlighted the anti-cancer properties of PRIMA-1/APR-246, in a large panel of human malignancies. Among these tumor diseases, the sensitivity to the cytotoxic effects of PRIMA-1/APR-246 varied. To illustrate these variations, the calculated IC50 (concentration of a drug that causes 50% growth inhibition) represent a first element, varying from 0.75 µM to 200 µM according to the tested models (Table 1). Besides the IC50, the evaluations of the PRIMA-1/APR-246 cytotoxicity demonstrated that it was dose-dependent [11,13,27,63], time-dependent [32,39,56,60,61], but also influenced by cellular confluence [19], hypoxia [59,66] or, more widely, the cellular context, as illustrated by the variable sensitivities of several cell lines established from the same patient with different tumor samples [11,31,57]. In addition, the anti-tumor effects of PRIMA-1 and APR-246 appeared mediated by changes in cell cycle progression, with an accumulation of cells in the G0/G1 phase [19,24,27,46,48,49,50,54,60], or in the G2 phase [8,20,26,32,48].

Importantly, no cytotoxic impact or limited cytotoxic impact has been reported when PRIMA-1/APR-246 was used with human normal cells, neither with normal hematological cells [11,36,63], nor with fibroblasts [8,26]. The data of tolerance mentioned in the in vivo studies are also reassuring: no change in animal weight or behavior has been reported in the mice treated with PRIMA-1 and APR-246—after either intra-tumoral injection, intra-venous administration, or intra-peritoneal treatment—using doses ranging from 20 mg/kg/day to 400mg/kg/day [8,19,27,28,29,36,37,38,40,42,48,55,56,58,62,65,69]. 

The main reported mechanism to explain the PRIMA-1/APR-246 induced cell death was the induction of apoptosis, evaluated using common methods such DNA fragmentation [8,28,50,65], morphologic modifications [35,52,68], APO2.7 (also named 7A6 antigen) expression [37], or Annexin V-binding [10,11,13,15,25,26,29,32,36,38,42,43,48,49,52,54,57,61,63]. Several studies demonstrated a global caspases activation [8,20,24,32,54], a specific cleavage of caspase 3 [14,26,27,28,29,30,32,36,37,40,48,50,62], or of the poly (ADR-ribose) polymerase (PARP) [25,27,36,37,42,43,45,48,50,52,54,57,60,63,66]. To confirm the essential role of caspases in PRIMA-1/APR-246 induced cell death, caspase inhibitors have been used, with a reduction, or in some cases, a suppression of the induced apoptosis [10,14,29,36,62,65]. The mitochondrial release of cytochrome C [10,14,68] or the activation of caspase-9 [32,43,68] attested to the possible implication of the intrinsic mitochondrial apoptosis, which could be independent of the p53-transcriptional activity [10,22]. By contrast, some authors did not conclude to apoptosis after a PRIMA-1/APR-246 exposure [17,18,41]. Indeed, although the tested doses were cytotoxic for non-small cell lung cancer [17], prostate cancer [18] or soft tissue sarcoma [41] cell lines, neither modification of the cell cycle [17,18], nor apoptotic bodies under microscope evaluation [18], nor PARP cleavage [17,41] were observed, regardless of the p53 status (wild type, mutated, or absent).

To demonstrate the reactivation of mutant-p53 under PRIMA-1/APR-246 treatment, several points have been evaluated. Firstly, a physical interaction between PRIMA-1/APR-246 and the p53 protein has been demonstrated. Lambert et al. (2009) [20] reported that both PRIMA-1 and APR-246 were converted in compounds, as MQ (methylene quinuclidinone), that reacted covalently with thiol groups of mutant, as well as wild-type p53. In particular, a pocket between loop L1 and sheet S3 of the p53 core domain, involving the Cys124 residue located at the center of the pocket, has been identified as a possible target of PRIMA-1 [70]. Secondly, the effects on the p53 protein in mutant-p53 cells treated with PRIMA-1/APR-246, were assessed. The results were controversial considering the level of p53 protein expression, (upregulated [15,30,50], or not [8,26,27,29,32,37,38]), as well as for the level of p53 protein phosphorylation at the Ser15 residue, used as a surrogate marker of p53 activation (phosphorylation [15,25,42,66], or not [8]). Similarly, the use of conformation-specific antibodies showed a shift of the p53 conformation toward a correctly folded protein [18,28,65], or not [54]. At last, mutant p53 protein appeared to be subjected to a nuclear translocation [12], with a shift toward the cells nucleoli [16,19,23]. Thirdly, the activity of p53 in cells with mutant-p53 treated with PRIMA-1/APR-246 has been explored. Few studies tested the specific DNA binding capacity of treated mutant-p53, concluding with an improvement [15,46,65], or not [19], of the p53-DNA interaction. The restoration of transcriptional activity of mutant p53 has also been assessed. p53-targeted genes implicated in cell cycle control, such as p21, has been described as upregulated [12,26,27,28,31,38,39,43,45,49,57,65], or not [17,19,22,32,37,41,47,50,54]. Concerning apoptosis genes, the results were also controversial with PUMA (p53 upregulated modulator of apoptosis), activated in many studies [15,29,43,47,48,57,68], but not in others [17,19,42,50,54], and with Bax (activated [15,26,27,31,32,38,39,47,48], or not [22,29,50]), but not with NOXA(Latin for damage), for which the activation seemed more consensual [22,36,37,42,43,47,48,57,68], although one exception [50] was reported. In addition, the assessment of inhibitors of p53 gene activation, such as MDM2, revealed the same discordant results (upregulation [27,31,32,65], or not [22]). Fourthly, the capacity of PRIMA-1/APR-246 to restore p53 activity has been questioned through its dependency to the mutant status of p53. This dependency has been tested directly, introducing mutant-p53 protein previously treated with PRIMA-1 into cells without p53, using the Chariot protein transfer reagent: this introduction induced cell death, G2 cell cycle arrest, phosphorylation at Ser15, expression of Bax, PUMA, and Noxa, improvement of specific DNA binding, and/or caspases activation [20]. The mutant-p53-dependency of PRIMA-1/APR-246 has also been evaluated indirectly, comparing the effects of these compounds on mutant- or wild-type-p53 cell lines. Again, the conclusions differed according to the authors, about the cytotoxic effect (dependent of mutant-p53 [25,31,32,38,39,41,44,47,48,61,65,66], or not [30,37,42,43,50,52,54,56,60]), about the pro–apoptotic effect (dependent of mutant-p53 [15,25,32,38,42,45,48,49,50], or not [10,29,36,37,52,54,57]), or about the transcriptional activator effect (dependent of mutant-p53 [12,14,15,22,25,26,31,38,39,47,48,49,61,65], or not [29,37,42]). Looking for homogeneous subgroups within these contradictory results, in order to define specific features, we have questioned the cancer type. Although mutant-p53-dependency appeared to be verified in five studies for breast cancer [12,14,15,45,61] and four studies for thyroid cancer [31,38,39,47], the conclusions must be nuanced because of several results were obtained using the same cell lines and by the same team ([12,14,15], and [38,39], respectively). On the contrary, the mutant-p53-independency of PRIMA-1/APR-246 effects in myeloma cells were verified by three different teams, using varied cell lines [36,37,54].

Finally, it clearly appeared that PRIMA-1/APR-246 are tumor suppressor molecules, inducing apoptosis by the caspases activation in varied mutant-p53 cells. If the proof of its mutant-p53 reactivation property has also been made in several cancer models, the variability of the results incites to look forward other elements likely to influence the effects of PRIMA-1/APR-246, as a cell content dependency [57], or unexpected cytotoxic mechanisms.

## 3. Unexpected Effects of PRIMA-1/APR-246 Driving New Hypothetical Mechanisms of Action 

The possibility of effects of PRIMA-1/APR-246 that were different from apoptosis and mutant-p53 reactivation has been underlined by the use of cell lines without p53 (p53-null) [8,10,19,20,22,24,25,27,31,34,37,40,41,42,48,49,52,54,56,57,65], or with a knock-down of p53 (p53-KD) [15,19,20,22,23,27,31,32,33,35,36,37,42,48,53,54,57]. 

**Figure 1:**
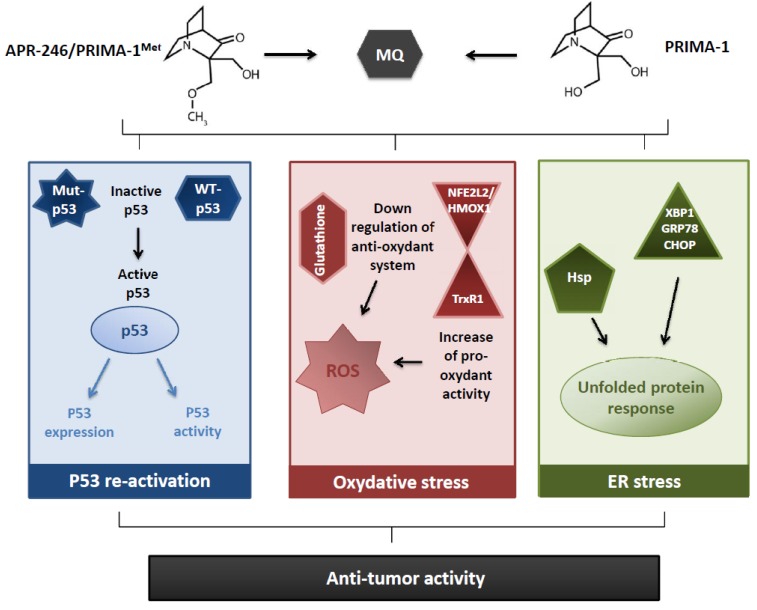
Main reported mechanisms of action of PRIMA-1and APR-246, leading to an anti-tumor activity.

Indeed, an absence, or a decrease, of cytotoxic [8,13,19,20,27,29,48,65], apoptotic [10,24,25,29,32,42,48,57], or transcriptional activator effects [15,20,24,25,27,29,31,48,57] have been observed in p53-null or p53-KD cells, after PRIMA-1/APR-246 exposure, and demonstrated a p53–dependency of both molecules in the induction of antitumor effects. Anyway, the finding of the same impacts, sometimes improved, in p53-null or in p53-KD cells (with cytotoxic [11,36,37,41,42,43,54,56], apoptotic [11,36,37,48,54], and transcriptional effects [19,36,37]) triggered the research of unexpected antitumor mechanisms (Figure 1).

First, the impact of PRIMA-1/APR-246 on cell redox balance has been assessed. Indeed, the role of the reactive oxygen species (ROS) in the PRIMA-1/APR-246 anti-tumor activity appeared since 2009 [20], with a global consensus between the authors: an increase of the amount of ROS [20,34,37,52,53,56,59,62] and a decrease of glutathione cellular content [37,40,53,62] have been widely reported under PRIMA-1/APR-246. The importance of ROS production in the PRIMA-1/APR-246-induced cell death was highlighted using a ROS scavenger, N-acetyl cysteine (NAC), which antagonist with PRIMA-1/APR-246 and inhibit its effect on cell proliferation [20,32,37,41,52,53] and apoptosis [20,32,37,52,66]. However, the conclusions from the NAC experiments should be analyzed keeping in mind the possible adduct formation between PRIMA-1 or APR-246 and NAC, decreasing the bioavailability and the efficacy of the two molecules. Glutathione metabolism seemed important in the PRIMA-1/APR-246 anti-tumor effects, as the use of buthionine-sulfoximine (BSO), an irreversible inhibitor of γ-glutamyl cysteine-synthase (γ-GCS), potentiated their anti-tumor activities [20,37,53,66]. These studies indicate that PRIMA synergize with BSO to induce cell death irrespective of p53 status. 

Likewise, the implication of SLC7A11, a key component of system xċ that imports cystine for the formation of glutathione, highlights the prominent role of glutathione content in APR-246 anti-tumor effect: SLC7A11 appears upregulated after APR-246 exposure [53], or proposed as a predictive biomarker for APR-246 sensitivity [62].

To understand the mechanisms of PRIMA-1/APR-246 associated to the redox status, several arguments have been proposed, concerning a decrease of the anti-oxydant response, or an increase of the pro-oxydant activity. The decrease of cellular glutathione appeared not caused by a decrease in the expression of the enzymes implicated in its production [37], but rather by the adducts formation between glutathione and MQ [40,62]. A greater sensitivity to oxidative stress was also explained through a downregulation or an inhibition of anti-oxidant enzymes—such as TrxR1 [34], Prx3, or GPx-1 [52]—and a dysregulation of the NFE2L2/HMOX1 axis [53]. On the contrary, the conversion of TrxR1 enzyme into a dedicated NADPH oxidase produced an increase oxidant activity [34]. The relationship between the redox balance and the p53 status remains discussed: several authors demonstrated the independency of the increase of ROS [41,56], or the decrease of glutathione [37], induced by APR-246 toward the p53 status. p53-independent mechanisms have been also proposed to elucidate the prooxidant effect of APR-246, based on the NFE2L2/HMOX1 axis [53] or on the TrxR1 enzyme [34]. On the contrary, Lambert et al. (2009) observed that APR-246 causes increased oxidation in a mutant p53-dependent manner [20]. The implication of mutant p53 proteins in the redox effects of APR-246 has been recently reported by Liu et al. (2017), considering that mutant p53 sensitized tumor cells to APR-246 induced oxidative stress, inhibiting the glutathione synthesis through the inhibition of system xċ [62]. Altogether, the most consensual results across cell lines and cell types (solid and hematologic cancers) are the production of ROS and the huge synergy with inhibitors of glutathione synthesis or cysteine transporter. Thus, PRIMA-1/APR-246,, by inducing massive ROS, can trigger a p53-dependent or independent cell death.

A second alternative mechanism of action of PRIMA-1/APR-246 concerned the unfolded protein response (UPR) (Figure 1). Indeed, this phenomenon, activated in response to an accumulation of unfolded or misfolded proteins in the endoplasmic reticulum (ER), also called ER stress, is initially dedicated to restore normal function of the cells by degrading misfolded proteins, and increasing the production of molecular chaperones, but can end up to apoptosis if the repairing mechanisms are overtaken. This notion has been evocated considering the upregulation of the heat shock proteins (Hsp), notably concerning the expression of Hsp 70 [16,21,36,54] and Hsp 90 [12], which increased under PRIMA-1/APR-246 treatment. Moreover, PRIMA-1/APR-246 were linked to the induction of several genes associated with the UPR [53], such as XBP1 [22], GRP78, or CHOP [54]. The importance of UPR in PRIMA-1 anti-tumor impacts has been highlighted by Teoh et al. (2016)[54], knocking-down CHOP, a specific factor mediating the ER stress-induced apoptosis, that led to a significant cytotoxicity decrease in p53-null cells.

PRIMA-1/APR-246 have also been associated to various tissular, cellular, and molecular mechanisms. At the tumor tissue level, PRIMA-1 induced the exposure of tumor epithelial cell anionic phospholipids, reduced the blood vessel density and the blood perfusion in breast cancer xenografts [28]. In two non-malignant pathologies, the ectrodactyly, ectodermal dysplasia, and cleft lip/palate (EEC) syndrome, and the squamous metaplasia, APR-246 restored, partially, a normal epithelial organization and differentiation in keratinocytes derived from EEP patients [71,72], or in human conjunctival tissues used as a squamous metaplasia model [73]. In cancer disease, PRIMA-1 and APR-246 may induce cell death through autophagy in breast cancer and sarcoma cells [33,41]. Senescence was also reported in one glioblastoma cell line exposed to APR-246 [60]. In human normal erythrocytes, PRIMA-1 induced eryptosis, a suicidal cell death, independent of any transcription activation [74]. PRIMA-1 also favored a partial differentiation of thyroid cancer cells, restoring the expression of natrium-iodine symporter (NIS) and thyroglobulin [31]. At the molecular level, different potential targets for PRIMA-1 and APR-246 have been screened. Initially, p53 family members have been explored: Rökaeus et al. (2010) [24] demonstrated a mutant-p63-dependency for APR-246 effects on cell proliferation, apoptosis, transcriptional activation in p53-null cells, and less clearly, a mutant-p73-dependency. Of note, the restoration of wild-type p63 functions in mutant-p63 cells were confirmed by studies on the EEC syndrome [71,72]. The role of p73 has been also assessed, using knocking-down models, and revealed that its suppression decreased the pro-apoptotic effects of PRIMA-1/APR-246 [36,43,54], and limited the expression of the PRIMA-1-induced ER stress markers [54]. Anyway, the implication of p73 was not confirmed by Messina et al. (2012), which demonstrated no effect of PRIMA-1 on thyroid cancer cells without p53, but with wild-type p73 [31]. Finally, several other studies used knocking-down experiments to demonstrate some mechanisms that could be involved in the PRIMA-1/APR-246 induced cell death. In particular, they reported that NOXA [36,37], microRNA-34a [25], microRNA-29a [55], MEK (Mitogen-activated protein/extracellular signal-regulated kinase kinase) [56], and c-myc [55] were linked to the anti-tumor effects of PRIMA-1/APR-246.

All these unexpected mechanisms of action of PRIMA-1/APR-246 multiplied the possible uses of these compounds for malignant, and non-malignant, pathologies. This wide action spectrum is still enlarged combining them with other treatments.

## 4. PRIMA-1 and APR-246 in Combination with Other Anti-Cancer Therapies

According to the cancer types, PRIMA-1/APR-246 have been associated to chemotherapy, radiotherapy, or targeted therapy. 

Basically, the chemotherapy drugs which lead to DNA damages and interfere with DNA synthesis were supposed to trigger p53 activation, and thus, to synergize with PRIMA-1/APR-246 in mutant-p53 cells. Several therapeutic agents have been associated to PRIMA-1/APR-246 in cell cultures, reporting additive or synergic effects. Among the pyrimidine analogs, a synergic anti-tumor effect of the treatment combination has been proven for 5-FU in head and neck [26], lung [8], oesophageal cancer [48], whereas, for gemcitabine, the association showed a synergic effect in ovarian cells [40], but an additive one in pancreatic cells [32], as well as for aracytin, in acute myeloid leukemia (AML) cells, where the time schedule and a pre-exposure to APR-246 seemed important [30]. For the purine analogs, the use of fludarabin with PRIMA-1/APR-246 had additive or synergic effects according to the tested B-CLL (Chronic lymphocytic leukemia) [11] or AML (Acute myeloid leukemia cell lines) [30], without any cross-resistance between PRIMA-1 and fludarabin [13]. Several intercalating agents demonstrated an increased cell toxicity when associated to PRIMA-1/APR-246, as doxorubicin in breast cancer (enhanced effect [12,45]), lung cancer (enhanced effect [8], or synergic effect [17]), thyroid cancer (enhanced effect [31,47]), pancreatic cancer (enhanced effect [67], or synergic effect [32]), myeloma (additive effect [36]), ovarian cancer (synergic effect and restoration of sensitivity in resistant cell line [40]). In this context, epirubicin had also synergic anti-tumor effects in esophageal adenocarcinoma cell lines [48], and daunorubicin in AML primary cultures [30]. Similarly, with topo-isomerase inhibitors, the results of their association with PRIMA-1/APR-246 varied as a synergic effect was obtained in lung, colon and osteosarcoma cell lines with camptothecin [8], but not in pancreatic cancer with irinotecan [32]. Platinum salts were also combined with PRIMA-1/APR-246, in particular cisplatin. A sensitization to cisplatin was observed with in thyroid cancer cells [31,47], a synergic cell toxicity was proven in colon [8], lung [8], head and neck cancer [26], oesophageal [62], pancreatic [32], and ovarian cancers. Besides, it has been reported that PRIMA-1/APR-246 restored sensitivity to cisplatin-resistant cell lines [35,40,51]. Anti-tumoral efficacy of these DNA-targeted associations has also been tested in vivo, for APR-246+5FU in esophageal cancer xenografts [48], for PRIMA-1/APR-246+Cisplatin 5FU in lung cancer [8], esophageal cancer [48], and ovarian cancer [40] xenografts. Independently of DNA damages, taxanes combined with PRIMA-1/APR-246 provided various results. PRIMA-1/APR-246 enhanced the anti-tumor effects of paclitaxel in lung cancer and osteosarcoma cell lines [8], and showed an additive effect with docetaxel in breast cancer cells [61], or a synergic one with taxol in head and neck cancer cells [26], whereas no sensitization was observed in thyroid cancer cells with taxol [31] or in ovarian cancer cells with docetaxel [40]. Among the spindle poisons, a synergic anti-tumor effect was demonstrated combining APR-246 to vinblastine in colon cancer cells [8], and to epirubicin in esophageal cancer cells [48]. Lastly, the associations of APR-246 with dexamethasone enhanced the cytotoxic effect in Waldenström cells [43] and in myeloma cell lines or xenografts [36]. In pre-clinical studies, 3-BrPA, a halogenated pyruvate derivative and an alkylating agent, depleting the cellular ATP pool and inhibiting glycolysis, has been associated to PRIMA-1. The association led to an enhanced anti-proliferative effect in mutant KRAS (Kirsten rat sarcoma viral oncogene homolog) lung cancer and melanoma cells [59], and in mutant-p53 bladder cancer cells [44].

As the ionizing radiation causes DNA damage, and, indirectly, a p53-activation, the association of PRIMA-1 or APR-246 with radiotherapy seems promising, although it remains little explored. The only study which focused on this combination reported a decrease of the surviving fraction, and of the clonogenic survival when prostate cancer cells were exposed to APR-246 for 24 h, with an irradiation occurring five hours after the beginning of APR-246 treatment [18]. APR-246 sensitized to irradiation the mutant-p53, and p53-null cells, but had no impact on wt-p53 cells. The mechanism of the radiosensitization of p53-null cells remained unexplained and could implicate the oxidative stress.

PRIMA-1 and APR-246 have been also associated to several targeted therapies, with a current clinical use or at a pre-clinical stage. Combined with the PARP-inhibitor, olaparib, APR-246 sensitized lung cancer cell lines to the targeted therapy, independently of p53 status [50]; besides, the combination restored the sensitivity to olaparib in mutant-p53 cells that were previously olaparib–resistant [50]. In breast cancer cell lines, the combination had a cytotoxic synergic effect in mutant p53 cells [61]. With the mTOR inhibitor, rapamycin, APR-246 had a cytotoxic synergic effect in a mutant-p53 AML cell line and in primary cultures [53]. With BRAF (v-Raf (Rapidly Accelerated Fibrosarcoma) viral oncogene homolog B) enzyme inhibitor, vemurafenib, APR-246 overcame acquired resistance to vemurafenib in melanoma cell lines and in xenografts, with a cytotoxic and proapototic synergic effect [58]. Strikingly and similarly, p53 reactivation by APR-246 also broke intrinsic and acquired resistance and synergized with the MEK inhibitor pimasertib to induce massive apoptosis in NRAS-mutant melanoma cells with wild-type or mutant-p53, identifying MITF/Bcl-2 as a key mechanism underlying resistance of mutant-NRAS melanoma cells to apoptosis by MEK inhibitors and propose clinically relevant drug combinations able to prevent or reverse it [64]. Combined with a tyrosine kinase inhibitor, erlotinib, PRIMA-1 synergized in mutant-p53 head and neck cancer [26] and pancreas cancer cells [32]. The anti-tumoral effect of PRIMA-1/APR-246 was enhanced when combined to the proteasome inhibitor, bortezomib, in mutant-p53 pancreas cancer cells [32], in wt-p53 Waldenström cells [43] and in myeloma cells, independently of p53 status, with a restoration to sensitivity in bortezomib-resistant cells [54]. According to the essential role of ROS production and glutathione content in PRIMA-1/APR-246 efficacy, associations between inhibitors of glutathione synthesis or cysteine transporter and PRIMA-1/APR-246 appear particularly relevant: thus, an inhibitor of the system x_ċ_, (cystine/glutamate antiporter), sulfasalazine, had a synergic anti-tumor effect with APR-246 in mutant p53 oesophageal adenocarcinoma cells and xenografts [62]. Considering targeted therapies in pre-clinical development, an enhanced cytotoxic effect has been observed in p53-mutant cancer cell cultures when PRIMA-1/APR-246 was combined to tunicamycin (ER stress inducer) in myeloma cells [54], or to wortmannin (PI3K inhibitor) in AML cells [53]. A synergic effect was found for nutlin-3 (MDM2 inhibitor) associated with PRIMA-1 in pancreatic cancer cells [32]. In vivo, an increase of the anti-tumor impacts has been observed when PRIMA-1 was associated to Deazaneplanocin A (a negative regulator of polycomb group actions that inhibits histone methyltransferase activity) in mutant-p53 thyroid cancer xenografts [38], and with 2aG4 (a monoclonal anti-body that binds specifically to the surface of tumor blood vessels and disrupts tumor vasculature) in breast cancer xenografts [28].

Altogether, these multiple efficient associations between PRIMA-1 or APR-246 and anti-cancer treatments make conceivable to treat many malignant diseases, and in particular, tumor sub-types, currently associated with poor prognosis because of genetic profile (mutant p53, KRAS, or BRAF) or acquired resistance to treatment (doxorubicin, cisplatin, olaparib, bortezomib, or vemurafenib).

## 5. Conclusions, Perspectives, and Clinical Impacts

In conclusion, PRIMA-1 and APR-246 appeared as important molecules with an anti-tumor effect in many cancer types. Its main cellular mechanism of action is the induction of apoptosis, mediated by the caspase activation. PRIMA-1 as well as APR-246 triggers an upregulation of genes involved in cell cycle control and apoptosis in mutant-p53 and wild-type p53 cancer cells. Anyway, 15 years after their discovery, it clearly appears that PRIMA-1/APR-246 have also p53-independent effects, as oxidative and ER stress, which emphasize their efficacies and extend their possible clinical uses, on tumor cells, independently of the p53 status. Combined with chemotherapies, ionizing radiations or targeted therapies, PRIMA-1 and APR-246 could offer new perspectives to treat the more aggressive tumor sub-types such as mutant-cKIT metastatic melanoma, HPV (Human papillomavirus)-positive head and neck squamous cell carcinoma, and anaplastic thyroid cancer. The first-in-human study (NCT00900614) that demonstrates, clinically, a good tolerance to the drug and a favorable pharmacokinetic profile, and biologically, an increased apoptosis with upregulation of p53 target genes, concludes to the safety of APR-246 use in hematologic malignancies and prostate cancer [75]. Three clinical trials are currently recruiting, with the objectives to test the safety and efficacy of APR-246 treatment in advanced oesophageal carcinoma (NCT02999893), high grade serous ovarian cancer (NCT02098343), and mutant p53 hematologic myeloid malignant disease (NCT03072043). Finally, a phase I/II study to investigate the safety and clinical activity of APR-246 in combination with a BRAF inhibitor in patients with mutant-BRAF unresectable metastatic melanoma resistant to anti-BRAF/anti-MEK inhibitors is starting.

## Figures and Tables

**Table 1:** Published studies since 2002 in Pubmed, reporting an anti-tumor effect (anti-proliferative and/or apoptotic effect) of PRIMA-1 or APR-246 in malignant disease, using established human cell lines, primary cultures or xenografts.

Reference	Molecule	Cancer Type	Reported IC_50_ (µmol/L)	Established Cell Line				Primary Culture (n)			Xenograft
				Wild-Type 53	Mutant p53	p53 Null	p53 KD	Wild-Type p53	Mutant p53	p53 Null	
**[7]**	PRIMA-1	Colorectal, lung, ovary, burkitt lymphoma, osteosarcoma	(0.75–65)	HCT116, HDF, IARC-171 EW36, Seraphin, KH39, Caki1	A461, SW480, Ramos, BL60, CW678, BL41, TK-10, KRC/Y+ (Saos-2-273, H1299-175, SKOV-175, SKOV-273, SKOV-175-22/23) ^a^	H1299, Saos-2, SKOV	HCT116				Yes
**[10]**	PRIMA-1	Lung (non-small cell)		H460	H23	H1299					
**[11]**	PRIMA-1	B cell chronic leukemia						9 ^b^		5 ^c^	
**[12]**	PRIMA-1	breast	(51–122)	MCF7	MDA-MB-231, GI-101A						
**[8]**	PRIMA-1 and APR-246	Colorectal, lung, osteosarcoma	PRIMA-1: (14–24) APR-246: (9–19)	HCT116	SW480+ (Saos-2-273, H1299-175) ^a^	H1299, Saos-2	HCT116				Yes
**[13]**	PRIMA-1	Acute myeloid leukemia						52 ^b^		8 ^c^	
**[14]**	PRIMA-1	Breast		MCF7	DA-MB-231						
**[15]**	PRIMA-1	Breast		MCF7	MDA-MB-231, GI-101A		MDA-MB-231, GI-101A				
**[16]**	PRIMA-1	Colorectal, lung, breast, osteosarcoma		MCF7, U205	SW480 + (H1299-175) ^a^	H1299					
**[17]**	PRIMA-1	Lung (non-small cell)	(60–175) *	A549	LX1, SKMes						
**[18]**	APR-246	Prostate		22RV1	DU145	PC3					
**[19**]	PRIMA-1	Hepato-cellular carcinoma			Mahlavu, PLC5/PRF/5 + (Hep3B-249, Hep3B-248) ^a^	Hep3B	PLC5/PRF/5				Yes
**[20]**	APR-246	Colorectal, lung, osteosarcoma, lymphoma	MQ: (14.8;20.6)	HCT116 + (H1299, BL41) ^d^	SBL41 + (Saos-2-273, H1299-175) ^a^	H1299, Saos-2	HCT116				
**[21]**	APR-246	Colorectal, lung, breast, lymphocyte		MCF7	SW480 + (H1299-175) ^a^	H1299					
**[22]**	APR-246	Colon, lung, osteosarcoma		HCT116 + (Saos-2) ^d^	SW480 + (Saos-2-273, H1299-175, HCT-116-248) ^a^	H1299, Saos-2	HCT116				
**[23]**	PRIMA-1	Breast			MDA-MB-231		MDA-MB-231				
**[24]**	APR-246	Colorectal, lung, osteosarcoma	(17–27) *			H1299, Saos-2-TA-p63γ, H1299-TA-p73α, H1299-TA-p73β, H1299-TA-p63γ	HCT116				
**[25]**	PRIMA-1	Lung		A549	H211, H1155	H1299					
**[26]**	PRIMA-1	Head and neck		JHU-028	UMSCC-22a, JHU-029, Fadu						
**[27]**	APR-246	Lung (small cell)		(CCD32Lu) ^e^	DMS456, DMS406, DMS273, DMS153, DMS114, DMS92, DMS79, DMS53, NCIH69, MAR24h, MAR86MI, GLC28, GLC26, GLC19, GLC16, GLC14, GLC3, GLC2 + (MDA-MB-231) ^f^	H1299	DMS273, DMS53, GLC16				Yes
**[28]**	PRIMA-1	Breast			BT-474, HCC-1428						Yes
**[29]**	APR-246	Melanoma		AA, FM88	C8161 + (M21) ^g^						Yes
**[30]**	APR-246	Acute myeloid leukemia	Mean = 5		KBM3			25	7		
**[31]**	PRIMA-1	Thyroid	(10–75) *	TPC-1	BC-PAP, Hth-74, FTC-133, C-643, 8305-C, FF-1	SW1736	Hth-74	Data not found			
**[32]**	PRIMA-1	Breast		MCF7	SKBR3 + (MCF7) ^a^						
**[33]**	PRIMA-1	Breast		MCF7, MRC5 + (HCT116) ^j^	MDA-MB-231, DLD-1		T1 + (HCT116) ^j^				
**[34]**	APR-246	Lung, osteosarcoma, burkitt lymphoma		(BL41) ^d^	BL41 + (Saos-2-273, H1299-175) ^a^	H1299, Saos-2					
**[35]**	PRIMA-1	Ovary			A2781cp		A2781cp				
**[36]**	APR-246	Myeloma	Cell line: (5–20) * Primary culture: (4–30) *	MM1S, H929	LP1, U266, 8266	8266R5	MM1S, U266	6		3 ^c^	Yes
**[32]**	PRIMA-1	Pancreas	(65–70)	Capan-2	PANC-1, BxPC-3		PANC-1				
**[37]**	APR-246	Myeloma	(3–200)	XG6, XG3, XG7, BCN, NAN9, H929, MDN, MM1S, AMO	NAN10, SKMM2, U266, XG1, XG11, XG5, 8226, JIM3, LP1, OPM2, XG2 + (NAN3, KMN1) ^h^	JJN3, KMS11, NAN1, L363	XG6, H929, XG5	16 ^b^		7 ^c^	Yes
**[38]**	PRIMA-1	Thyroid		TPC-1, K1, IHH4	FTC-133, WRO, 8505C, C-643, BC-PAP						
**[39]**	PRIMA-1	Thyroid	(28.7–128.5)	K1, IHH4	C-643, BC-PAP						
**[40]**	APR-246	Ovary	(11–37)	A2780, A2780cis, A2780adr	OVCAR-3, A2780-CP20, IGROV-1/CDDP + (H1770, H1975, H596, H378) ^i^	(H1417) ^i^		1	4		Yes
**[41]**	APR-246	Sarcoma	(7.9–26.1)	IB139 + (HCT116) ^j^	IB130, IB134, IB138 + (HT-29) ^j^	IB136, IB117					
**[42]**	APR-246	Colorectal	(7–58.6)	HCT116, RKO, LOVO	DLD-1, SW480, SW620, Colo320, Caco2, HT29		HCT116				Yes
**[43]**	APR-246	Waldenström	(10–30)	BCWM-1	MWCL-1			2			
**[44]**	PRIMA-1	Bladder		RT4	T24, T24-X						
**[45]**	PRIMA-1	Breast		MCF-10A	T47D, MDA-MB-468						
**[46]**	PRIMA-1	Lung (non-small cell)		A549	NSCLL-N6						
**[47]**	PRIMA-1	Thyroid		WRO	FTC-133						
**[48]**	APR-246	Oesophageal	(10–100) *	(NES) ^k, l^	FLO-1, Eso26, OE19, OANC1, JH-EsoAd1, SKGT4, OE33, OACM5.1 + (H1299) ^a,i^	OACP4C, TE7 ^m^ + (H1299) ^i^	OACM5.1, OANC1, FLO-1, OE19, JH-EsoAd				Yes
**[49]**	PRIMA-1	Prostate		LNCap + (PC3) ^d^	DU145 + (PC3) ^a^	PC3					Yes
**[50]**	APR-246	Lung (non-small cell)	(9.56–29.35)	A549	1975, H2228, H596						
**[51]**	APR-246	Ovary	(5.2–56)					1	9 ^n^		
**[52]**	APR-246	Ovary	(2.6–20.1)	NOS2, TOV21G, A2780	NOS3, OVCAR-3, CAOV-3, OV-90, ES-2	SKOV-3					
**[53]**	APR-246	Acute myeloid leukemia		(HCT116) ^j^	KBM3 + (HCT116) ^a,j^		(HCT116) ^j^	5			
**[54]**	PRIMA-1 and APR-246	Myeloma	PRIMA-1: (16.3–88.9) APR-246: (2–24.5)	H929 + (XG6, KMS18) ^h^	U266, 8226, KMS28	KMS11 + (JJN3) ^m^	H929				
**[55]**	APR-246	Myelome		MM1S, H929	U266, 8226, LP1			5			Yes
**[56]**	APR-246	Colorectal		HCT 116, LOVO	SW480, DLD-1, HT29	HCT116	SW480, DLD-1				Yes
**[57]**	APR-246	Ewing sarcoma		TC252	STA-ET-7.2, RDES, IARC-EW2, RM82, SK-ES1, STA-ET-2.2 + (MDA-MB-468) ^f^	A673, SK-N-MC	STA-ET-7.2		(+3) ^o^		
**[58]**	APR-246	Melanoma		MM070, MM034, MM050,MM133, MM032, MM043, MM074-R, MM029, MM074, MM054	Sk-MEL-28, MM164						Yes
**[59]**	PRIMA-1	Melanoma		MelJuso, C8161 + (A549) ^i^							
**[60]**	APR-246	Glioblastoma	(60–100)	U87MG, U87/EV, U87/MGMT	T98/EV, T98/shRNA, U138, LN-18, A172						
**[61]**	PRIMA-1 and APR-246	Breast	PRIMA-1: (1.4–15.1) APR-246: (0.9–31.1)	UACC812, Hs878T(i8), ZR-75-1,MCF7,BT474 + (MCF12A, MCF10A) ^k^	Hs878T(i8)2, HCC70, Hs578T, CAL-85-1, HCC1143, BT474, HCC1937, HDQ-P1, BT20, JimT1, Cama1, T47D, BT549,MDA-MB-468, MDA-MB-453						
**[62]**	APR-246	Oesophageal		(NES) ^k,l^	FLO-1, Eso26, OE19, OANC1, JH-EsoAd1, SKGT4, OE33, OACM5.1 + (H1299) ^a,i^	OACP4C, TE7 ^m^ + (H1299) ^i^	OACM5.1, OANC1, FLO-1, OE19, JH-EsoAd				Yes
**[63]**	PRIMA-1	Acute promyelocytic leukemia			NB4						
**[64]**	APR-246	Melanoma		MM161, MM057, MM165, MM052, MM167	MM125						

* IC_50_ estimated on graphs; ^a^ Cell lines genetically modified to introduce a mutant-p53 gene; ^b^ Without hemizygous p53-deletion or with less than 50% of hemizygous 53-deletion; ^c^ With hemizygous p53-deletion; ^d^ Cell lines genetically modified to introduce a wild-type p53 gene; ^e^ Normal fibroblast cell line; ^f^ Breast cancer cell line; ^g^ Cell line with mutant-p53 that it behaves as a wild-type-p53; ^h^ Cell line presenting a mixed status: wild-type/mutant p53; ^i^ Lung cell line; ^j^ Colon cell line; ^k^ Immortalized cell lines; ^l^ Normal oesohageal cell line; ^m^ Mutated cell lines classified as p53null because no protein expression was detected; ^n^ Cell lines with p53 missense mutations (n = 7) or nonsense mutations (n = 2); ^o^ The three cell lines were from the same patient, with several origin (primary tumor or metastasis); ^p^ Gastric cell line.
